# Synthesis of 5-(aryl­methyl­idene­amino)-4-(1*H*-benzo[*d*]imidazol-1-yl)py­rimi­dine hybrids: synthetic sequence and the mol­ecular and supra­molecular structures of two inter­mediates and three final products

**DOI:** 10.1107/S2053229623003728

**Published:** 2023-05-05

**Authors:** Daniel E. Vicentes, Ricaurte Rodríguez, Justo Cobo, Christopher Glidewell

**Affiliations:** a Departamento de Química Inorgánica y Orgánica, Universidad de Jaén, 23071 Jaén, Spain; b Campus Universitario, Universidad de Ciencias Aplicadas y Ambientales (UDCA), Calle 222, No. 55-37, Bogotá, Colombia; c Departamento de Química, Universidad Nacional de Colombia, Cuidad Universitaria, Carrera 30, No. 45-03, Edificio 451, Bogotá, Colombia; d School of Chemistry, University of St Andrews, St Andrews, Fife KY16 9S, United Kingdom; Wilfrid Laurier University, Waterloo, Ontario, Canada

**Keywords:** synthesis, py­rimi­dine, heterocyclic hybrid, NMR spectroscopy, crystal structure, mol­ecular structure, mol­ecular conformation, hydro­gen bonding, supra­molecular assembly

## Abstract

A concise and versatile synthesis of 5-(aryl­methyl­idene­amino)-4-(1*H*-benzo[*d*]imidazol-1-yl)py­rimi­dines has been developed, starting from 4-(1*H*-benzo[*d*]imidazol-1-yl)py­rimi­dines, and we report here the synthesis and spectroscopic and structural characterization of three such products, along with those of two inter­mediates in the reaction pathway. The inter­mediates 4-[2-(4-chloro­phen­yl)-1*H*-benzo[*d*]imidazol-1-yl]-6-meth­oxy­py­rimi­dine-2,5-di­amine, (II), and 4-[2-(4-bromo­phen­yl)-1*H*-benzo[*d*]imidazol-1-yl]-6-meth­oxy­py­rimi­dine-2,5-di­amine, (III), crystallize as the isostructural monohydrates C_18_H_15_ClN_5_O·H_2_O and C_18_H_15_BrN_5_O·H_2_O, respectively, in which the components are linked into complex sheets by O—H⋯N and N—H⋯O hydro­gen bonds. In the product (*E*)-4-meth­oxy-5-[(4-nitro­benzyl­idene)amino]-6-[2-(4-nitro­phen­yl)-1*H*-benzo[*d*]imidazol-1-yl]pyrimidin-2-amine, which crystallizes as a 1:1 solvate with dimethyl sulfoxide, C_25_H_18_N_8_O_5_·C_2_H_6_OS, (IV), inversion-related pairs of the py­rimi­dine component are linked by N—H⋯N hydro­gen bonds to form cyclic centrosymmetric *R*_2_^2^(8) dimers to which pairs of solvent mol­ecules are linked by N—H⋯O hydro­gen bonds. (*E*)-4-Meth­oxy-5-[(4-methyl­benzyl­idene)amino]-6-[2-(4-methyl­phen­yl)-1*H*-benzo[*d*]imidazol-1-yl]pyrimidin-2-amine, C_27_H_24_N_6_O, (V), crystallizes with *Z*′ = 2 and the mol­ecules are linked into a three-dimensional framework structure by a combination of N—H⋯N, C—H⋯N and C—H⋯π(arene) hydro­gen bonds. The analogous product (*E*)-4-meth­oxy-5-[(4-chloro­benzyl­idene)amino]-6-[2-(4-methyl­phen­yl)-1*H*-benzo[*d*]imidazol-1-yl]pyrimidin-2-amine, C_26_H_21_ClN_6_O, (VI), crystallizes from dimethyl sulfoxide in two forms: one, denoted (VI*a*), is isostructural with (V), and the other, denoted (VI*b*), crystallizes with *Z*′ = 1, but as an unknown solvate in which the py­rimi­dine mol­ecules are linked by N—H⋯N hydro­gen bonds to form a ribbon containing two types of centrosymmetric ring.

## Abstract 2

A versatile synthesis is reported for 5-(aryl­methyl­idene­amino)-4-(1*H*-benzo[*d*]imidazol-1-yl)py­rimi­dines and structures are reported for three examples, one of them in two crystal forms, as well as for two inter­mediates in the synthetic sequence. A diverse range of hydro­gen-bonding patterns leads to supra­molecular assemblies ranging from finite zero-dimensional aggregates to three-dimensional framework structures.

## Introduction

The benzimidazole unit has been shown to be an important heterocyclic fragment present in a large number of com­pounds with broad biological activity, including anti­microbial and anti­tumour activity (El-Gohary & Shaaban, 2017 ▸). In addition, amino­py­rimi­dines are important building blocks for the synthesis of new heterocyclic systems (Abdul-Rida *et al.*, 2017 ▸), and they are also considered to constitute an important pharmacophoric fragment (Loving *et al.*, 2009 ▸), because of the wide biological activities that com­pounds containing this unit have shown, including anti-HIV activity (Al-Masoudi *et al.*, 2016 ▸) and cyclin-dependent kinase 2 (CDK2) inhibitory activity (Cortese *et al.*, 2016 ▸).

Mol­ecules which include both benzimidazole and amino­py­rimi­dine nuclei have been studied against some cancer cell lines, yielding inter­esting results that motivate the synthesis of this type of hybrid structures. This is the case for a series of novel fused pyrimido–benzimidazole systems reported re­cently, where one of the structures showed an IC_50_ value less than 2 µ*M* against the neuroblastoma SK-N-BE(2)-C and Kelly cell lines (Gadde *et al.*, 2023 ▸).

Non-fused py­rimi­dine–benzimidazole hybrids have also exhibited promising results for anti­tumour activity in human cancer cell lines (Sana *et al.*, 2021 ▸). A recent report has attributed the cytotoxicity of py­rimi­dine–benzimidazole hybrids to the presence of meth­oxy groups on the arene rings linked to the py­rimi­dine core, while the presence of electron-withdrawing groups seems to eliminate anti­cancer activity (Ismail *et al.*, 2022 ▸). However, any attempt to predict, prior to experimental evaluation, the effects of substituent variation in the products reported here would, perforce, be largely speculative and thus will not be pursued in this article.

We have recently reported the synthesis and the mol­ecular and supra­molecular structures of a set *N* ^5^-aryl­methyl-6-meth­oxy-4-(2-aryl-1*H*-benzo[*d*]imidazol-1-yl)py­rimi­dine-2,5-di­amines, where the two pendent aryl residues are identical, as they are both introduced in the reaction of *N* ^4^-(2-amino­phen­yl)-6-meth­oxy­py­rimi­dine-2,4,5-tri­amine with an aryl aldehyde in a 1:2 molar ratio (Vicentes *et al.*, 2019 ▸). Because of the biological importance of both the 2-amino­py­rimi­dine residue (Koroleva *et al.*, 2010 ▸; Jadhav *et al.*, 2021 ▸) and the benzimidazole unit (Singh *et al.*, 2013 ▸; Wu *et al.*, 2022 ▸), whether alone or in combination, in the search for new biological targets (Sana *et al.*, 2021 ▸), we have now explored the combination of different aryl residues linked to the 5-amino group.

We report here an extension of the py­rimi­dine–benzimidazole hybrid systems reported previously (Vicentes *et al.*, 2019 ▸), in which the *N* ^5^-methylaryl-6-meth­oxy-4-(2-aryl-1*H*-benzo[*d*]imidazol-1-yl)py­rimi­dine-2,5-di­amine precusors (A) (see Scheme 1) are subjected to de­benzyl­ation effected by ammonium hexa­nitratocerate(IV) (CAN) to produce the 6-meth­oxy-4-(2-aryl-1*H*-benzo[*d*]imidazol-1-yl)py­rimi­dine-2,5-di­amines (I)–(III) (Scheme 1) for use as inter­mediates in the derivatization at the 5-amino group. When the corresponding reaction was attempted using the type (A) precursor having *X* = *Y* = NO_2_, no de­benzyl­ation was observed, but instead the reaction produced a complex mixture from which only (*E*)-4-meth­oxy-5-[(4-nitro­benzyl­idene)amino]-6-[2-(4-nitro­phen­yl)-1*H*-benzo[*d*]imidazol-1-yl]pyrimidin-2-amine, (IV), could be isolated in pure form as a 1:1 solvate with di­methyl sulfoxide, in a yield of only 15%.

When com­pound (I) was condensed with 4-methyl­benz­al­de­hyde, the product (*E*)-4-meth­oxy-5-[(4-methyl­benzyl­idene)amino]-6-[2-(4-methyl­phen­yl)-1*H*-benzo[*d*]imidazol-1-yl]pyrimidin-2-amine, (V), was formed in 74% yield and straightforwardly crystallized in the solvent-free form from a mixture of ethyl acetate and hexane. However, the corresponding reaction with 4-chloro­benz­al­de­hyde gave, after crystallization, a mixture of two crystalline forms of (*E*)-4-chloro-5-[(4-methyl­benzyl­idene)amino]-6-[2-(4-methyl­phen­yl)-1*H*-benzo[*d*]imidazol-1-yl]pyrimidin-2-amine, one denoted (VI*a*), which is isostructural with (V), together with a second form, denoted (VI*b*), which is a solvate of unknown constitution. The structure of com­pound (I) has already been reported (Vicentes *et al.*, 2019 ▸) and we report here the mol­ecular and supra­molecular structures of com­pounds (II)–(VI). 
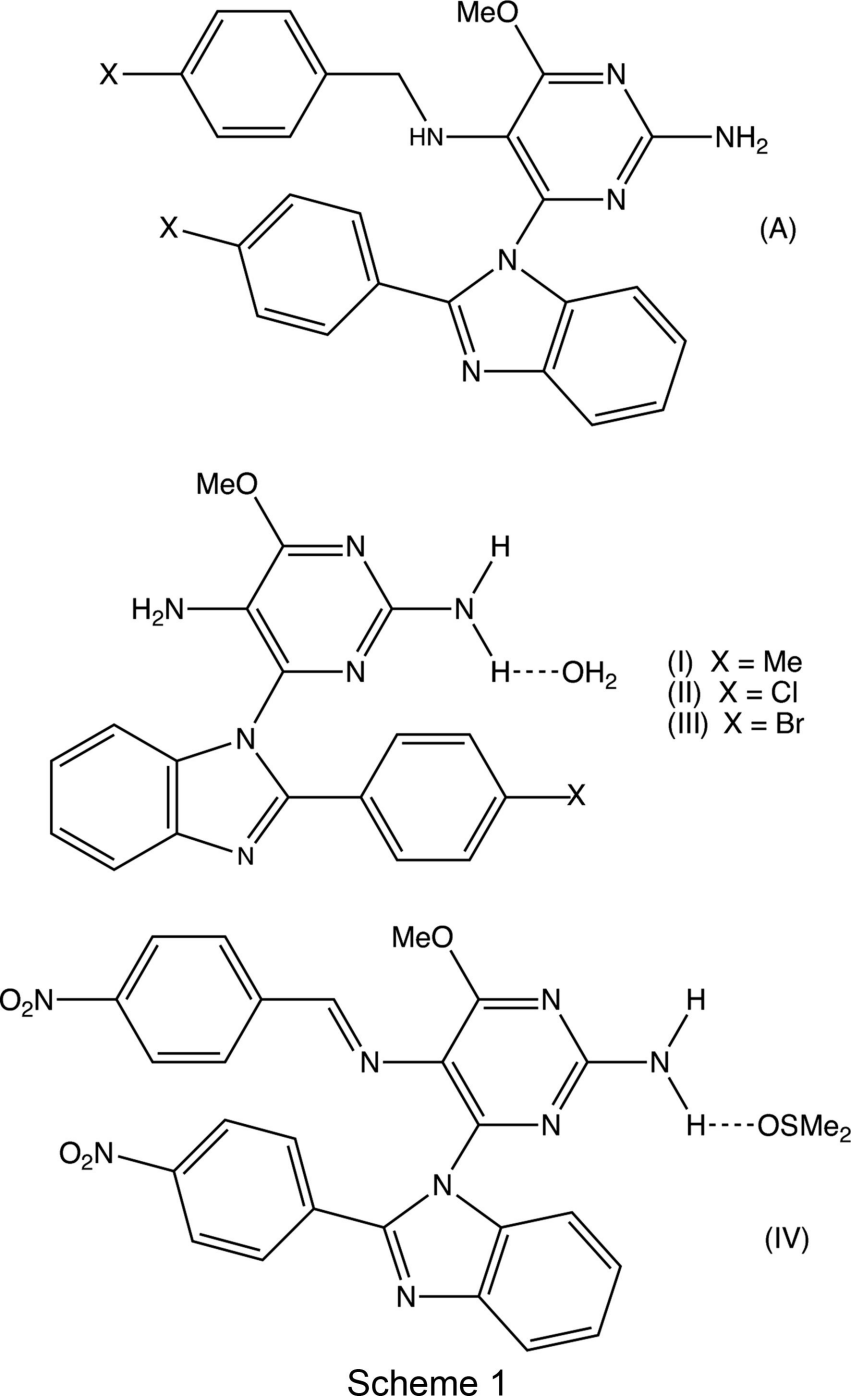


## Experimental

### Synthesis and crystallization

The atom labelling is based throughout on the IUPAC chemical names, with the immediate substituents on the py­rimi­dine ring labelled according to their location; thus, N21, N41, N51 and O61, with appropriate modifications when *Z*′ = 2, and with the rest of the substituent labels following the IUPAC name.

All of the signals in the ^1^H and ^13^C NMR spectra listed below were assigned using one-dimensional DEPT-135 ^13^C spectra and two-dimensional COSY, HSQC and HMBC spectra.

**Figure 1:**
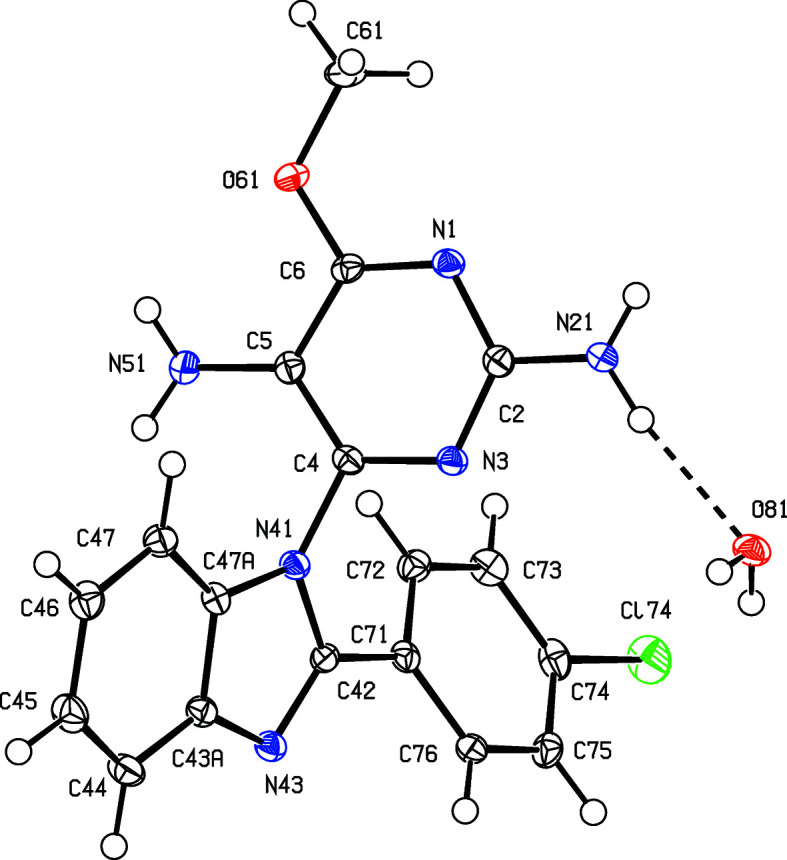
The two independent components in the structure of com­pound (II), showing the atom-labelling scheme and the hydro­gen bond (drawn as a dashed line) within the selected asymmetric unit. Displacement ellipsoids are drawn at the 50% probability level.

The precursors of type (A) and the inter­mediate (I) (see Scheme 1) were prepared using previously described methods (Vicentes *et al.*, 2019 ▸). In the NMR listings given below, the atom labelling for com­pounds (II)–(IV) and (VI) follows that used in Figs. 1 ▸–3 ▸ ▸ and 6, and the labelling for com­pound (V) follows that for (VI).

For the synthesis of com­pounds (II)–(IV), a solution of ammonium hexa­nitratocerate(IV) (0.69 g, 1.5 mmol) in a mixture of aceto­nitrile and water (3:1 *v*/*v*, 50 ml) was added to a solution of the appropriate precursor (A) [0.5 mmol; 0.22 g for (II) and 0.26 g for each of (III) and (IV)] in aceto­nitrile (10 ml); the resulting mixtures were then stirred for 2 h at 273 K. A saturated solution of sodium carbonate (15 ml) was then added and the aceto­nitrile was removed under reduced pressure. The residue was exhaustively extracted with ethyl acetate and the combined organic extracts were washed with water and then dried over anhydrous sodium sulfate. The solvent was removed under reduced pressure and the crude solid products purified by column chromatography on silica gel (0.040–0.063 mm) using a mixture of ethyl acetate and hexane (3:2 *v*/*v*) as the eluent.

Compound (II): colourless solid, yield 54%, m.p. 510 K (decomposition). IR (ATR, cm^−1^): 3494, 3399, 3303, 3188, 2922, 1606, 1562, 1467, 1450, 1403, 1261, 1241, 1092, 1011, 798, 739. NMR (DMSO-*d*_6_): δ(^1^H, 400 MHz) 7.79 (*ddd*, *J* = 8.0, 1.3, 0.7 Hz, 1H, H44), 7.62 (*d*, *J* = 8.8 Hz, 2H, H72, H76), 7.28 (*d*, *J* = 8.8 Hz, 2H, H73, H75), 7.25 (*dd*, *J* = 4.0, 1.5 Hz, 1H, H45), 7.21 (*dd*, *J* = 7.2, 1.3 Hz, 1H, H46), 7.15 (*ddd*, *J* = 7.8, 1.4, 0.7 Hz, 1H, H47), 4.56 (*s*, 2H,NH_2_), 3.97 (*s*, 3H, OCH_3_), 3.06 (*s*, 2H, NH_2_); δ(^13^C, 101 MHz) 162.33 (C6), 155.03 (C2), 150.94 (C42), 143.39 (C43*A*), 140.29 (C4), 136.25 (C74), 135.24 (C47*A*), 129.95 (C72, C76), 129.04 (C73, C75), 128.57 (C71), 124.12 (C46), 123.65 (C45), 120.30 (C44), 117.03 (C5), 110.86 (C47), 54.75 (OCH_3_). HRMS (ESI–QTOF) *m*/*z* found 367.1069, [*M* + H]^+^ requires for C_18_H_15_ClN_6_O, 367.1069.

Compound (III): colourless solid, yield 48%, m.p. 508 K (decomposition). IR (ATR, cm^−1^): 3494, 3398, 3302, 3185, 1338, 1609, 1562, 1466, 1450, 1401, 1241, 1053, 1008, 832, 741. NMR (DMSO-*d*_6_): δ(^1^H, 400 MHz) 7.75 (*d*, *J* = 7.3 Hz, 1H, H44), 7.63 (*m*, 4H, H72, H73, H75, H76), 7.32–7.21 (*m*, 1H, H45, H46), 7.12 (*d*, *J* = 7.4 Hz, 1H, H47), 5.99 (*s*, 2H, NH_2_), 4.17 (*s*, 2H, NH_2_), 3.96 (*s*, 3H, OCH_3_); δ(^13^C, 101 MHz) 161.49 (C6), 154.72 (C2), 150.65 (C42), 142.74 (C43*A*), 138.91 (C4), 135.55 (C74A), 131.54 (C73, C75), 130.16 (C72, C76), 129.46 (C71), 123.33 (C46), 123.25 (C45), 122.67 (C74), 119.34 (C44), 116.92 (C5), 111.32 (C47), 54.03 (OCH_3_). HRMS (ESI–QTOF) *m*/*z* found 413.0541, [*M* + H]^+^ requires for C_18_H_15_BrN_6_O, 413.0545.

Compound (IV): yellow solid, yield 15%, m.p. 517 K (decomposition). IR (ATR, cm^−1^): 3449, 3338, 3230, 1638, 1559, 1527, 1448, 1339, 853, 743. NMR (DMSO-*d*_6_): δ(^1^H, 400 MHz) 8.66 (*s*, 1H, H57), 8.18 (*d*, *J* = 8.8 Hz, 2H, H53, H55), 8.13 (*d*, *J* = 9.0 Hz, 2H, H73, H75), 7.88–7.84 (*m*, 1H, H44), 7.82 (*d*, *J* = 9.1 Hz, 2H, H72, H76), 7.59–7.51 (*m*, 3H, H47, H52, H56), 7.49 (*s*, 2H, NH_2_), 7.41–7.27 (*m*, 2H, H45, H46), 4.01 (*s*, 3H, OCH_3_); δ(^13^C, 101 MHz) 164.07 (C6), 160.63 (C2), 156.79 (C57), 154.28 (C4), 150.82 (C74), 148.31 (C54), 147.57 (C42), 142.59 (C43*A*), 142.26 (C51), 136.55 (C71), 136.34 (C47*A*), 128.83 (C72, C73), 128.34 (C53, C56), 124.25 (46), 123.84 (C53, C55), 123.71 (C73, C75), 123.42 (C45), 119.81 (C44), 114.09 (C5), 112.24 (C47), 54.46 (OCH_3_). HRMS (ESI–QTOF) *m*/*z* found 511.1471, [*M* + H]^+^ requires for C_25_H_18_N_8_O_5_, 511.1471.

For the synthesis of com­pounds (V) and (VI) (Scheme 2), a mixture of com­pound (I) (0.17 g, 0.05 mmol) and the appropriate benz­al­de­hyde (0.7 mmol) [84 mg of 4-methyl­benz­al­de­hyde for (V) or 92 mg of 4-chloro­benz­al­de­hyde for (VI)] in acetic acid (3 ml) was stirred at ambient temperature for 1 h. The resulting precipitates were collected by filtration and washed first with an aqueous solution of sodium hydro­gen carbonate (10% *w*/*v*) and then with water. The crude solid products were then purified by column chromatography on silica gel (0.040–0.063 mm) using a mixture of ethyl acetate and hexane (3:2 *v*/*v*) as eluent. 
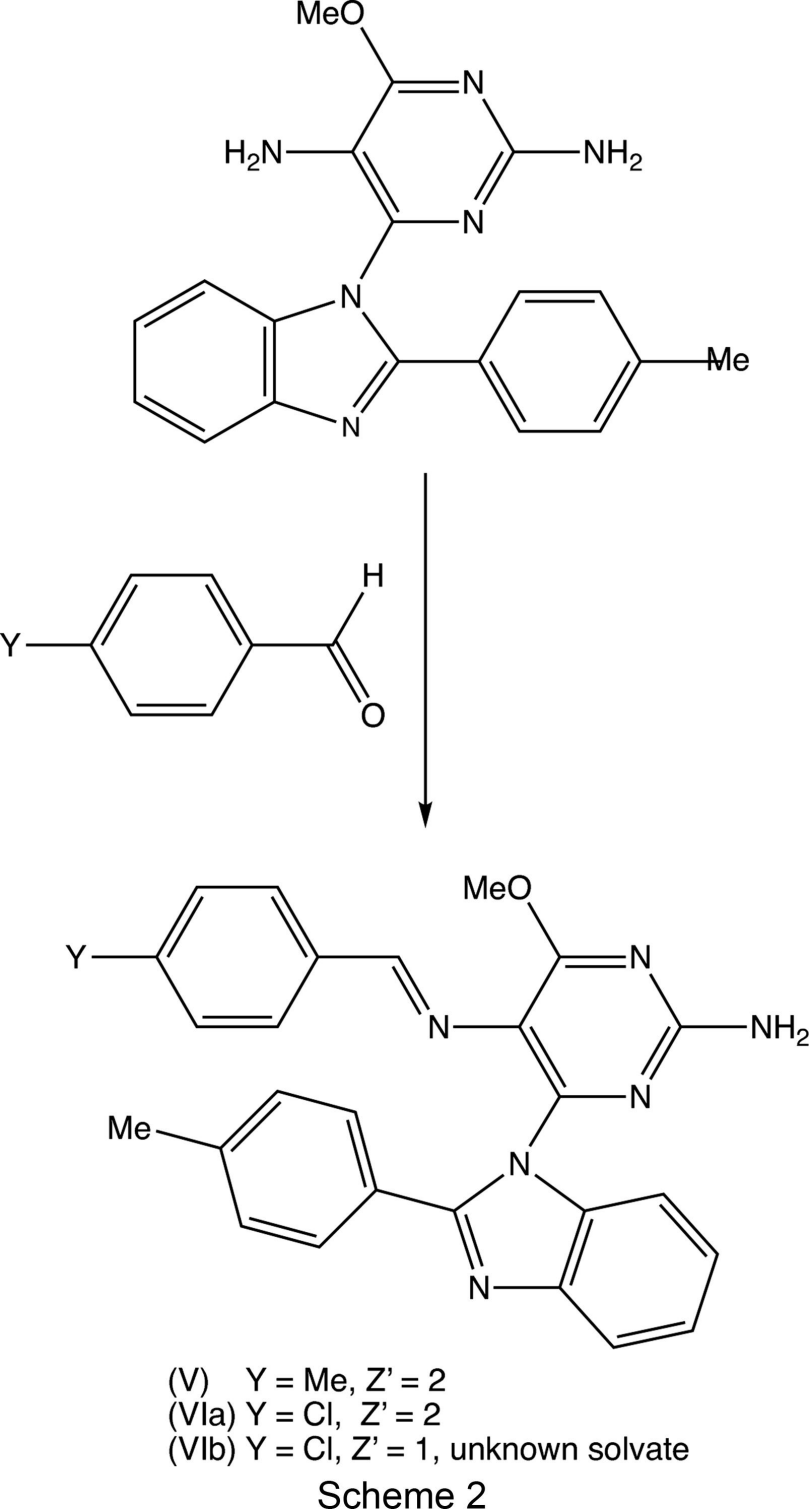


Compound (V): yellow solid, yield 74%, m.p. 491 K (decomposition). IR (ATR, cm^−1^): 3303, 3157, 1652, 1606, 1576, 1522, 1449, 1345, 1248, 1078, 1041, 817, 738. NMR (DMSO-*d*_6_): δ(^1^H, 400 MHz) 8.33 (*s*, 1H, H57), 7.73 (*d*, *J* = 6.9 Hz, 1H, H44), 7.45 (*d*, *J* = 8.2 Hz, 2H, H72, H76), 7.37 (*d*, *J* = 6.7 Hz, 1H, H47), 7.29–7.19 (*m*, 4H, H45, H46, H52, H56), 7.16–7.07 (*m*, 6H, H73, H75, H53, H55, NH_2_), 3.94 (*s*, 3H, OCH_3_), 2.26 (*s*, 3H, C77), 2.25 (*s*, 3H, C58); δ(^13^C, 101 MHz) 163.79 (C6), 160.29 (C2), 159.89 (C57), 152.97 (C42), 152.89 (C4), 142.67 (C43*A*), 140.92 (C34), 139.15 (C74), 136.35 (C47*A*), 134.01 (C28), 129.06 (C73, C75), 128.88 (C53, C55), 127.87 (C71), 127.73 (C52, C56), 127.66 (C72, C76), 122.92 (C46), 122.55 (C45), 119.03 (C44), 116.05 (C5), 111.45 (C47), 54.18 (OCH_3_), 21.02 (C77), 20.83 (C58). HRMS (ESI–QTOF) *m*/*z* found 449.2084, [*M* + H]^+^ requires for C_27_H_24_N_6_O, 449.2084.

Compound (VI): yellow solid, yield 95%, m.p. 493 K (decomposition). IR (ATR, cm^−1^): 3308, 3138, 1652, 1573, 1520, 1450, 1362, 1080, 1042, 821, 735. NMR (DMSO-*d*_6_): δ(^1^H, 400 MHz) 8.43 (*s*, 1H, H57), 7.76 (*d*, *J* = 6.8 Hz, 1H, H44), 7.45 (*d*, *J* = 8.2 Hz, 2H, H72, H76), 7.44–7.36 (*m*, 3H, H47, H53, H55), 7.34 (*d*, *J* = 8.6 Hz, 2H, H52, H56), 7.31–7.19 (*m*, 4H, H45, H26, NH_2_), 7.09 (*d*, *J* = 8.0 Hz, 2H, H73, H75), 3.97 (*s*, 3H, OCH_3_), 2.25 (*s*, 3H, CCH_3_); δ(^13^C, 101 MHz) 163.85 (C6), 160.18 (C2), 158.37 (C57), 153.78 (C4), 153.01 (C42), 142.70 (C43*A*), 139.21 (C74), 136.34 (C47*A*), 135.50 (C54), 135.42 (C51), 129.21 (C52, C56), 128.92 (C73, C75), 128.62 (C53, C55), 127,86 (C71), 127.64 (C72, C76), 123.01 (C46), 122.66 (C45), 119.10 (C44), 115.24 (C5), 111.54 (C47), 54.28 (OCH_3_), 20.85 (CH_3_). HRMS (ESI–QTOF) *m*/*z* found 469.1538, [*M* + H]^+^ requires for C_26_H_21_ClN_6_O, 469.1538.

Crystals of com­pounds (II)–(V) suitable for single-crystal X-ray diffraction were grown by slow evaporation at ambient temperature and in the presence of air from a solution in dimethyl sulfoxide for (II), (IV) and (V) or from a solution in methanol for (III), providing (II) and (III) as monohydrates, (IV) as a dimethyl sulfoxide (DMSO) solvate and (V) in the solvent-free form. A similar crystallization of (VI) from a solution in DMSO yielded two types of crystal, *i.e.* the more block-like solvent-free form (VI*a*) and the more plate-like solvate (VI*b*); no attempt was made to determine the relative qu­anti­ties of the two crystalline forms.

### Refinement

Crystal data, data collection and refinement details for com­pounds (II)–(VI) are summarized in Table 1 ▸. For (VI*b*), one reflection (010), which had been attenuated by the beam stop, and one bad outlier reflection ( 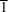 03) were omitted from the data set. All H atoms were located in difference maps. The H atoms bonded to C atoms were then treated as riding atoms in geometrically idealized positions, with C—H = 0.95 (alkenic and aromatic) or 0.98 Å (CH_3_), and with *U*_iso_(H) = *kU*_eq_(C), where *k* = 1.5 for the methyl groups, which were permitted to rotate but not to tilt, and 1.2 for all other H atoms. For the H atoms bonded to N or O atoms, the atomic coordinates were refined with *U*_iso_(H) = 1.2*U*_eq_(N) or 1.5*U*_eq_(O), giving the N—H and O—H distances shown in Table 3. For (VI*b*), conventional refinement converged only to *R*_1_ = 0.146 and *wR*_2_ = 0.3473. Examination of the structure of (VI*b*) at this point using *PLATON* (Spek, 2020 ▸) showed that the structure formed by the mol­ecules of (VI) enclosed a void centred at (0,0, 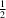 ), whose volume was *ca* 166 Å^3^ in a unit cell of total volume 1272.6 (2) Å^3^. The void thus occupies *ca* 13.0% of the total unit-cell volume, and there are a number of significant peaks in the difference map clustered within this void. The largest peak had a magnitude of 4.64 e Å^−3^ and further examination of this structure using the SQUEEZE procedure (Spek, 2015 ▸) indicated that the void contained around 43 electrons not hitherto accounted for. This number is consistent with the presence of one mol­ecule of dimethyl sulfoxide, but no convincing solvent model could be developed from the difference peaks within the void and hence the reflection data were subjected to the SQUEEZE procedure (Spek, 2015 ▸), and the resultant modified reflection file was used for the refinement reported here. The CIF describing the structure obtained before the SQUEEZE procedure was applied has been included in the supporting information.

## Results and discussion

Oxidation of the type (A) precursors having *X* = Cl or Br gave the products (II) and (III) (see Scheme 1) in exactly the same way as reported previously for the formation of (I) (Vicentes *et al.*, 2019 ▸). The formation of (I)–(III) presumably proceeds *via* the oxidation of the precursors to form the corresponding Schiff bases, which are hydrolysed to (I)–(III) during the subsequent work-up procedures. Accordingly, the formation of (IV), albeit in low yield, when *X* = NO_2_, was unexpected, as it might be expected that this Schiff base would be more susceptible to hydrolysis than those having *X* = Me, Cl or Br. Condensation of (I) with two representative substituted benz­al­de­hydes gave the required hybrid products (V) and (VI) in yields of 74 and 95%, respectively.

The new com­pounds (II)–(VI) reported here were all fully characterized by high-resolution mass spectrometry, by IR and ^1^H and ^13^C NMR spectroscopy, where the NMR spectra exhibited all of the expected signals, and by single-crystal X-ray diffraction. The crystallographic study confirmed fully the constitutions deduced from the spectra and, in addition, demonstrated the *E* configuration at the exocyclic C=N double bonds in (IV)–(VI), as well as providing information about the mol­ecular conformations in the solid state and about the supra­molecular assembly.

In the synthesis of the type (A) precursors (Vicentes *et al.*, 2019 ▸), the benzimidazole unit was constructed during the synthesis by condensation of an aldehyde with a py­rimi­dine-substituted benzene-1,2-di­amine. The ability to incorporate a variety of substituents into both of these components, as well as into the aldehydes used in the formation of the products (V) and (VI), thus offers the possibility of forming a large library of variants containing multiple and varied substituents.

**Figure 3:**
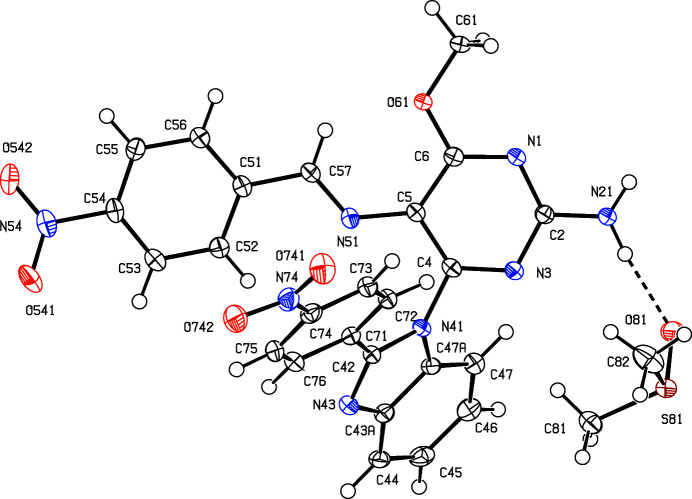
The two independent components in the structure of com­pound (IV), showing the atom-labelling scheme and the hydro­gen bond (drawn as a dashed line) within the selected asymmetric unit. Displacement ellipsoids are drawn at the 50% probability level.

**Figure 4:**
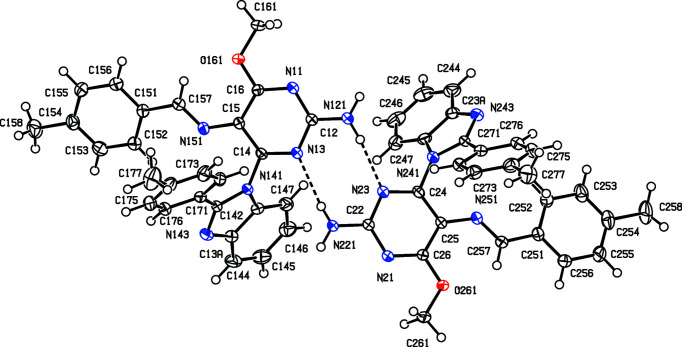
The two independent components in the structure of com­pound (V), showing the atom-labelling scheme and the hydro­gen bonds (drawn as dashed lines) within the selected asymmetric unit. Displacement ellipsoids are drawn at the 50% probability level.

**Figure 6:**
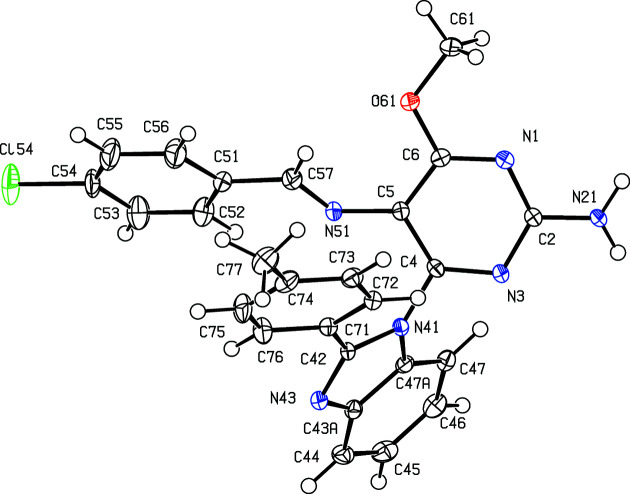
The mol­ecular structure of the *Z*′ = 1 form of com­pound (VI), denoted (VI*b*), showing the atom-labelling scheme. Displacement ellipsoids are drawn at the 50% probability level.

The inter­mediates (II) and (III) are isostructural (Table 1 ▸) with the methyl analogue (I) (Vicentes *et al.*, 2019 ▸) and they crystallize as monohydrates (Figs. 1 ▸ and 2 ▸). The product (IV) crystallizes as a stoichiometric solvate with dimethyl sulfoxide (Fig. 3 ▸), but the products (V) and (VI*a*), which are isostructural, crystallize in the solvent-free form with *Z*′ = 2 (Figs. 4 ▸ and 5 ▸). The second crystalline form of com­pound (VI), denoted (VI*b*) (Fig. 6 ▸), also crystallizes as a solvate, but no coherent model for the disordered solvent could be developed from the peaks in the difference map; accordingly, the SQUEEZE procedure (Spek, 2015 ▸) was applied to the data set for this com­pound before the final refinements (see Section 2.2). For each of (V) and (VI*a*), a search for possible additional crystallographic symmetry revealed none; however, the two independent mol­ecules in each of these com­pounds are related by an approximate, but noncrystallographic, twofold rotation axis (Figs. 4 ▸ and 5 ▸).

None of the py­rimi­dine components in com­pounds (II)–(VI) exhibits any inter­nal symmetry, as indicated by the key torsion angles (Tables 2 ▸ and 3 ▸), and hence all are conformationally chiral (Moss, 1996 ▸; Flack & Bernardinelli, 1999 ▸), but the space groups (Table 1 ▸) confirm that, in every case, equal numbers of the two conformational enanti­omers are present. For each of the products (IV)–(VI), the reference mol­ecules were selected to have a positive sign for the torsion angles N*x*3—C*x*4—N*x*41—C*x*42, where *x* = 1 or 2 for (V) and (VI*a*), and *x* = nil for (IV) and (VI*b*) (Table 3 ▸). On this basis, each product has a negative sign for the torsion angle N3—C4—N41—C47*A* in (IV) and (VI*b*) or N*x*3—C*x*4—N*x*41—C*x*47 in (V) and (VI*a*) (see Figs. 3 ▸–6 ▸ ▸ ▸). All of the pro­ducts have a negative sign for the torsion angle N*x*41—C*x*42—C*x*71—C*x*72 and, in each product, the magnitudes of the corresponding torsion angles are very similar (Table 3 ▸). Overall, the products (IV)–(VI) all have very similar mol­ecular structures but their crystallization characteristics are different as noted above and, as discussed below, their supra­molecular arrangements are also very different.

In the inter­mediates (II) and (III), the signs and magnitudes of the torsion angles N3—C4—N41—C42 and N3—C4—N41—C47*A* are effectively inter­changed compared with the corresponding angles in the products (IV)–(VI) (Tables 2 ▸ and 3 ▸, and Figs. 1 ▸–6 ▸ ▸ ▸ ▸ ▸). In effect, the orientation of the benzimidazole unit in (II) and (III) relative to the py­rimi­dine differs from that in the products (IV)–(VI) by a rotation of *ca* 180° about the C—N bond linking these two ring systems. Since the imino atom N43/N*x*43 is involved in inter­mol­ecular hydro­gen bonding in every com­pound apart from (IV) (Table 4 ▸), it is not easy to understand these orientational differences. In each of com­pounds (II)–(VI), the meth­oxy C atom is effectively coplanar with the adjacent py­rimi­dine ring, as indicated by the torsion angles involving these C atoms (Tables 2 ▸ and 3 ▸).

The inter­mediates (II) and (III) are isostructural with (I) (Vicentes *et al.*, 2019 ▸), and thus exhibit the same pattern of supra­molecular assembly, forming complex sheets built from a combination of O—H⋯N and N—H⋯O hydro­gen bonds. No additional comment is required except to note that the structure of com­pound (III) contains a fairly short inter­molecular Br⋯O contact whose dimensions are Br74⋯O61^i^ = 3.0972 (16) Å and C74—Br74⋯O61^i^ = 173.70 (9)° [symmetry code: (i) *x*, *y* − 1, *z*], so that the Br⋯O distance is shorter than the sum of the conventional van der Waals radii of 3.41 Å (Rowland & Taylor, 1996 ▸). However, the conventional radii are derived assuming no directional variation in the effective van der Waals radius, but detailed database analysis (Nyburg & Faerman, 1985 ▸) for nonbonded contacts involving halogen atoms bonded to C atoms indicates significant angular variation, with the effective radii diminishing as the contact angle approaches 180°, as here. On this basis, the sum of the effective van der Waals radii, 3.08 Å, differs little from the distance observed here, so that this contact in com­pound (III) should not be regarded as structurally significant.

**Figure 7:**
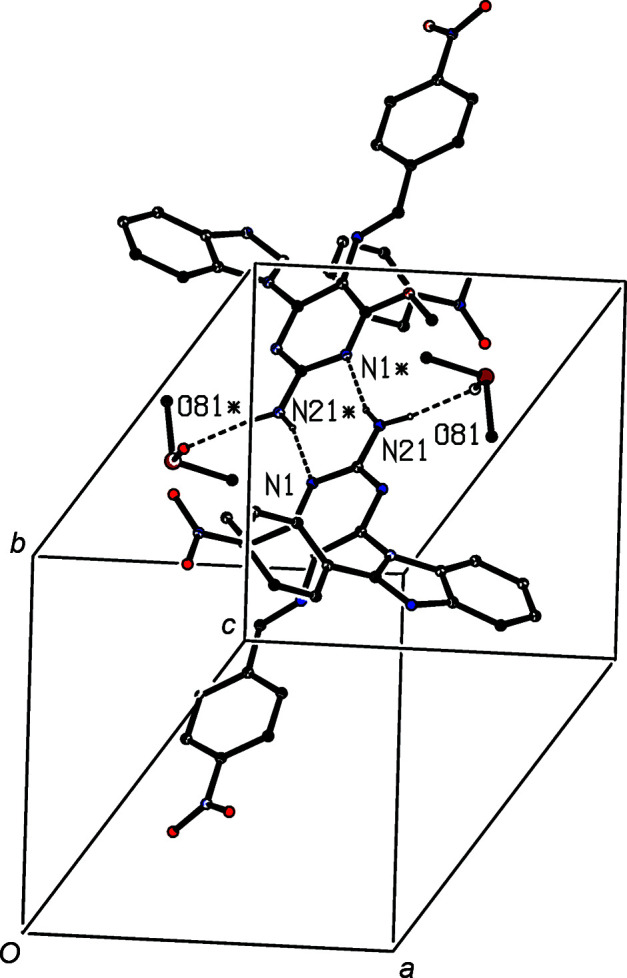
Part of the crystal structure of com­pound (IV), showing the formation of a centrosymmetric four-mol­ecule aggregate built from N—H⋯O and N—H⋯N hydro­gen bonds, which are drawn as dashed lines. For the sake of clarity, H atoms which are not involved in the motifs shown have been omitted. Atoms marked with an asterisk (*) are at the symmetry position (−*x* + 1, −*y* + 2, −*z* + 1).

For the product (IV), the supra­molecular assembly is very simple: inversion-related py­rimi­dine components are linked by N—H⋯N hydro­gen bonds to form a cyclic centrosymmtric 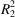 (8) dimer, to which inversion-related solvent mol­ecules are linked by N—H⋯O hydro­gen bonds (Fig. 7 ▸). There are no direction-specific inter­actions between the four-mol­ecule aggregates of this type.

**Figure 8:**
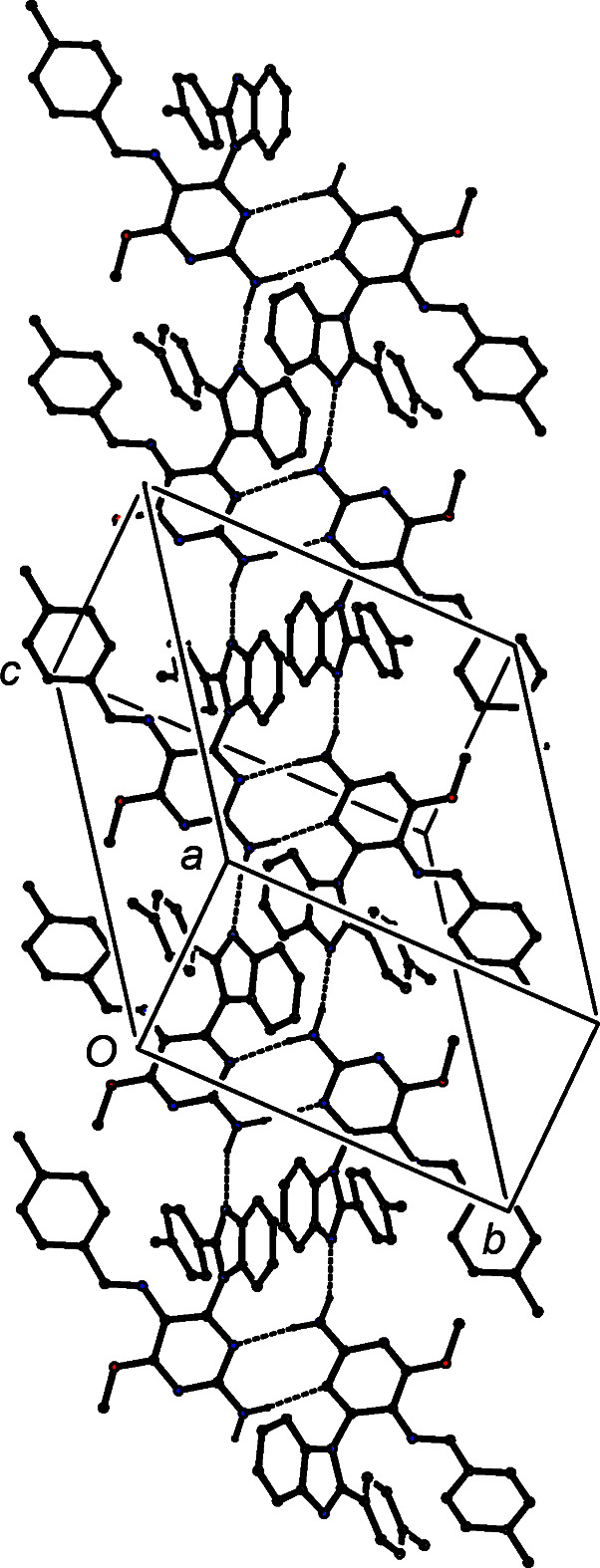
Part of the crystal structure of com­pound (V), showing the formation of a ribbon of alternating 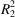 (8) and 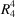 (16) rings running parallel to the [101] direction. Hydrogen bonds are drawn as dashed lines and, for the sake of clarity, H atoms bonded to C atoms have all been omitted.

**Figure 9:**
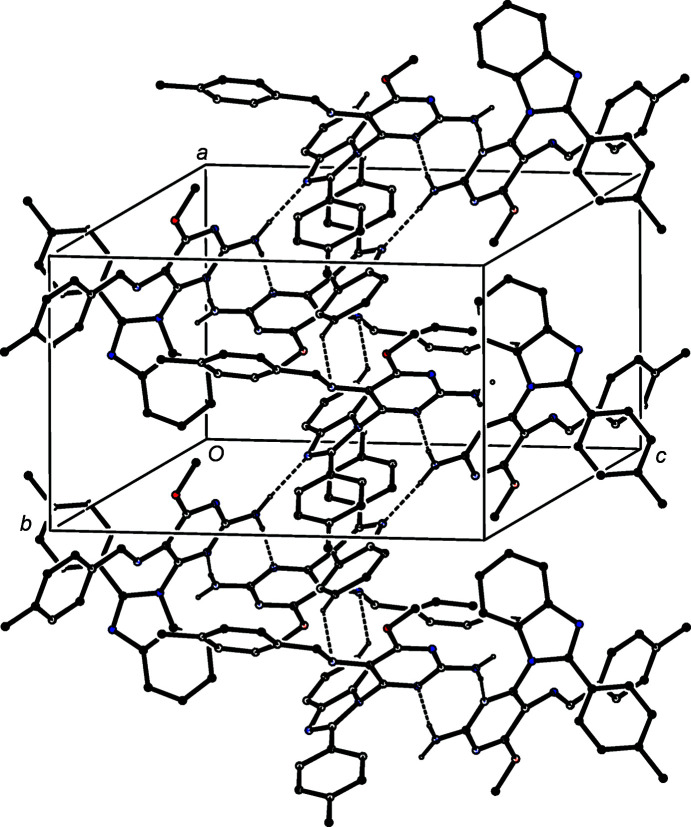
Part of the crystal structure of com­pound (V), showing the formation of a ribbon of alternating 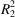 (8) and 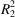 (16) rings running parallel to the [100] direction. Hydrogen bonds are drawn as dashed lines and, for the sake of clarity, H atoms bonded to C atoms but not involved in the motif shown have been omitted.

**Figure 10:**
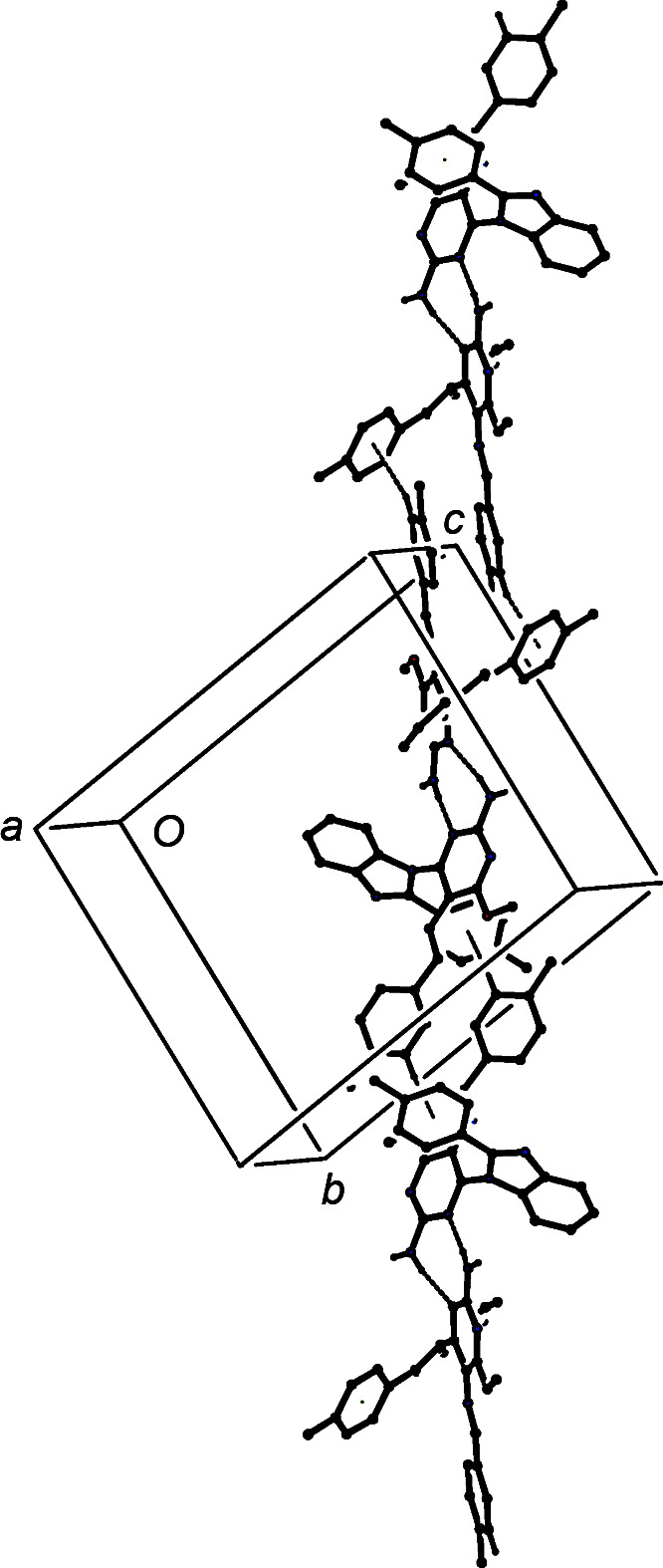
Part of the crystal structure of com­pound (V), showing the formation of a chain of rings along [12 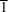 ] built from N—H⋯N and C—H⋯π(arene) hydro­gen bonds, which are drawn as dashed lines. For the sake of clarity, H atoms bonded to C atoms but not involved in the motif shown have been omitted.

In the isostructural products (V) and (VI*a*), there are eight independent hydro­gen bonds, four of the N—H⋯N type and two each of the C—H⋯N and C—H⋯π(arene) types (Table 4 ▸), which together link the mol­ecules into three-dimensional framework structures. The differences in the details of the C—H⋯N and C—H⋯π(arene) hydro­gen bonds involving the two independent mol­ecules confirms the lack of additional crystallographic symmetry. In the selected asymmetric units (Figs. 4 ▸ and 5 ▸), the two mol­ecules are linked by N—H⋯N hydro­gen bonds, and these dimeric units can be regarded as the basic building block for the three-dimensional assembly, which is readily analysed in terms of simple one-dimensional substructures (Ferguson *et al.*, 1998*a* ▸,*b* ▸; Gregson *et al.*, 2000 ▸). Two N—H⋯N hydro­gen bonds, having atoms N143 and N243 as the acceptors (Table 4 ▸), link the basic dimers into a ribbon of alternating 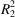 (8) and 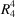 (16) rings running parallel to the [101] direction (Fig. 8 ▸). In the second substructure, the linking of the basic dimeric units by the two C—H⋯N hydro­gen bonds generates a ribbon of alternating 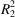 (8) and 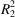 (16) rings running parallel to the [100] direction (Fig. 9 ▸). In the final substructure, the linkage of the dimers by two C—H⋯π(arene) hydro­gen bonds generates a chain of rings running parallel to the [12 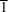 ] direction (Fig. 10 ▸). The combination of the chain motifs along [100], [101] and [12 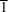 ] suffices to generate a three-dimensional framework structure.

**Figure 11:**
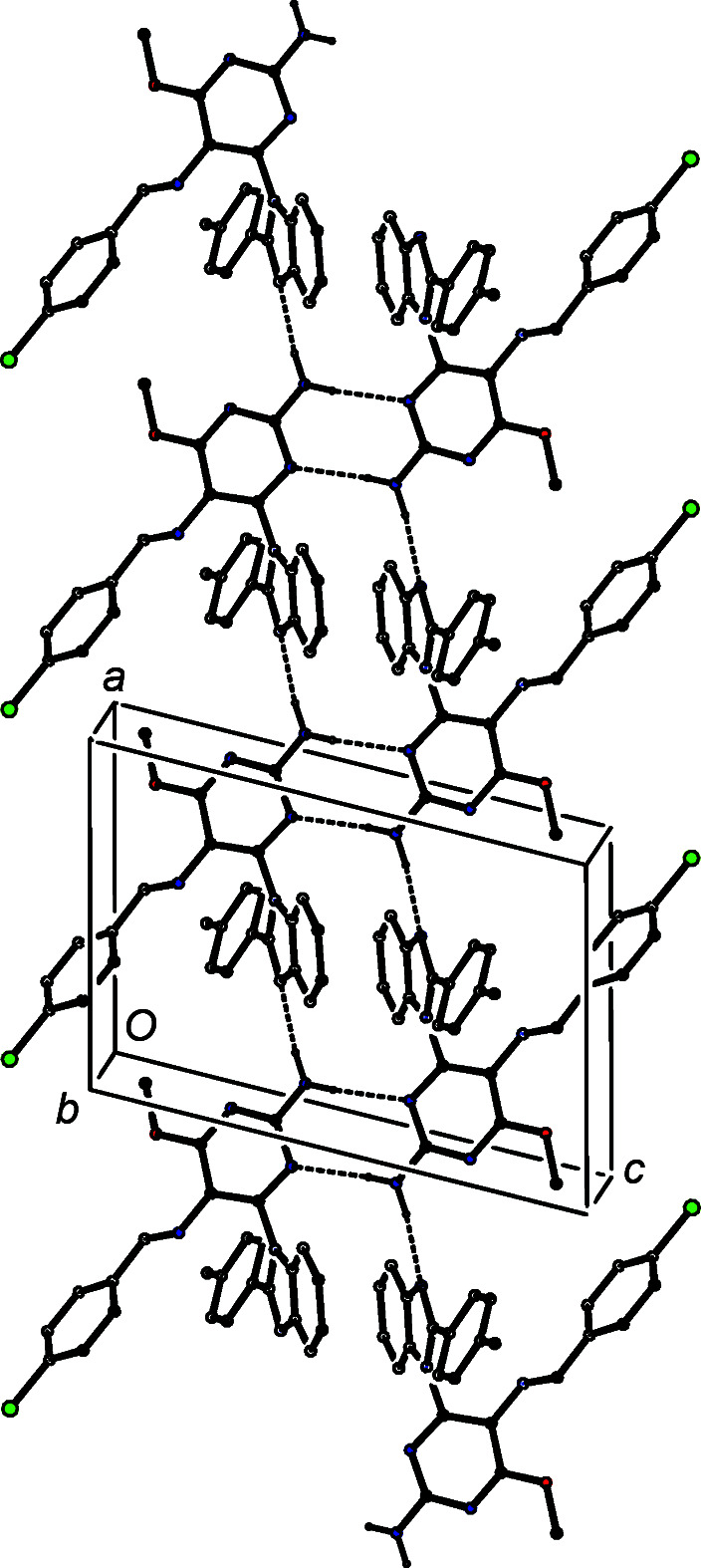
Part of the crystal structure of form (VI*b*), showing the formation of a ribbon of centrosymmetric 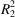 (8) and 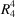 (16) rings running parallel to [100]. Hydrogen bonds are drawn as dashed lines and, for the sake of clarity, H atoms bonded to C atoms have all been omitted.

Two N—H⋯ N hydro­gen bonds link the mol­ecules of (VI*b*) into a ribbon of edge-fused centrosymmetric rings running parallel to [100], in which 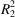 (8) rings (Etter, 1990 ▸; Etter *et al.*, 1990 ▸; Bernstein *et al.*, 1995 ▸) centred at (*n*, 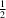 , 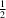 ) alternate with 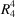 (16) rings centred at (*n* + 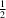 , 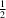 , 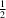 ) (Fig. 11 ▸), where *n* represents an integer in each case.

We have previously reported the structures of a wide range of multiply-substituted py­rimi­dines, but many of these carry either C-nitroso (Quesada *et al.*, 2002 ▸, 2004 ▸; Melguizo *et al.*, 2003 ▸) or C-formyl substituents (Cobo *et al.*, 2008 ▸), whose presence is associated with highly polarized electronic structures.

## Summary

We have developed a versatile and efficient synthesis of 5-(aryl­methyl­idene­amino)-4-(1*H*-benzo[*d*]imidazol-1-yl)py­rimi­dine hybrids based on simple starting materials and we have characterized three products and two inter­mediates spectroscopically (IR, ^1^H and ^13^C NMR, and HRMS) and have determined their mol­ecular and supra­molecular structures.

## Supplementary Material

Crystal structure: contains datablock(s) global, II, III, IV, V, VIa, VIb. DOI: 10.1107/S2053229623003728/dv3022sup1.cif

Structure factors: contains datablock(s) II. DOI: 10.1107/S2053229623003728/dv3022IIsup2.hkl

Structure factors: contains datablock(s) III. DOI: 10.1107/S2053229623003728/dv3022IIIsup3.hkl

Structure factors: contains datablock(s) IV. DOI: 10.1107/S2053229623003728/dv3022IVsup4.hkl

Structure factors: contains datablock(s) V. DOI: 10.1107/S2053229623003728/dv3022Vsup5.hkl

Structure factors: contains datablock(s) VIa. DOI: 10.1107/S2053229623003728/dv3022VIasup6.hkl

Structure factors: contains datablock(s) VIb. DOI: 10.1107/S2053229623003728/dv3022VIbsup7.hkl

Supporting information file. DOI: 10.1107/S2053229623003728/dv3022IIsup8.cml

Supporting information file. DOI: 10.1107/S2053229623003728/dv3022IIIsup9.cml

Supporting information file. DOI: 10.1107/S2053229623003728/dv3022IVsup10.cml

Supporting information file. DOI: 10.1107/S2053229623003728/dv3022Vsup11.cml

Supporting information file. DOI: 10.1107/S2053229623003728/dv3022VIasup12.cml

CIF describing the structure of (VI obtained before the SQUEEZE procedure was applied. DOI: 10.1107/S2053229623003728/dv3022sup13.txt

CCDC references: 2258812, 2258811, 2258810, 2258809, 2258808, 2258807

## Figures and Tables

**Figure 2:**
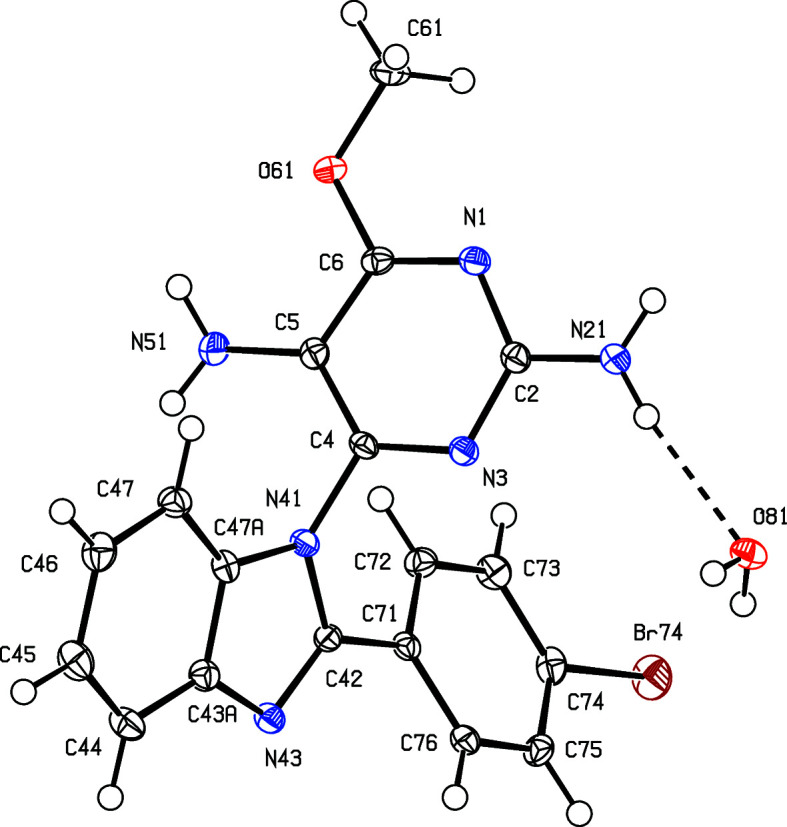
The two independent components in the structure of com­pound (III), showing the atom-labelling scheme and the hydro­gen bond (drawn as a dashed line) within the selected asymmetric unit. Displacement ellipsoids are drawn at the 50% probability level.

**Figure 5:**
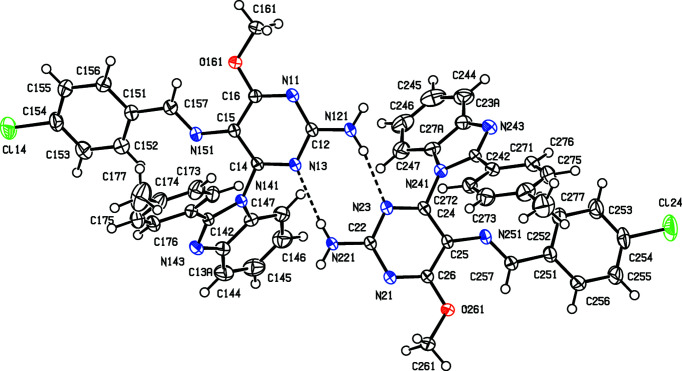
The two independent components in the structure of the *Z*′ = 2 form of com­pound (VI), denoted (VI*a*), showing the atom-labelling scheme and the hydro­gen bonds (drawn as dashed lines) within the selected asymmetric unit. Displacement ellipsoids are drawn at the 50% probability level.

**Table:** For all structures: triclinic, *P* 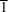 . Experiments were carried out at 100 K with Mo *K*α radiation using a Bruker D8 Venture diffractometer. Absorption was corrected for by multi-scan methods (*SADABS*; Bruker, 2016 ▸). H atoms were treated by a mixture of independent and constrained refinement.

	(II)	(III)	(IV)
Crystal data			
Chemical formula	C_18_H_15_ClN_6_O·H_2_O	C_18_H_15_BrN_6_O·H_2_O	C_25_H_18_N_8_O_5_·C_2_H_6_OS
*M*_r_	384.83	429.29	588.60
*a*, *b*, *c* (Å)	8.2156 (6), 11.0343 (7), 11.3968 (8)	8.1975 (7), 11.1963 (8), 11.3644 (10)	9.8192 (8), 10.2765 (7), 14.4096 (11)
α, β, γ (°)	107.980 (2), 109.725 (2), 98.541 (2)	107.938 (2), 109.866 (3), 98.683 (3)	71.718 (2), 74.872 (3), 88.786 (3)
*V* (Å^3^)	887.43 (11)	894.39 (13)	1329.87 (18)
*Z*	2	2	2
μ (mm^−1^)	0.24	2.33	0.18
Crystal size (mm)	0.22 × 0.20 × 0.14	0.15 × 0.11 × 0.08	0.19 × 0.15 × 0.10

Data collection			
*T*_min_, *T*_max_	0.907, 0.967	0.719, 0.830	0.905, 0.982
No. of measured, independent and observed [*I* > 2σ(*I*)] reflections	35335, 4071, 3487	50505, 4457, 3696	66738, 6115, 4641
*R*_int_	0.053	0.073	0.112
(sin θ/λ)_max_ (Å^−1^)	0.650	0.667	0.649

Refinement			
*R*[*F* ^2^ > 2σ(*F* ^2^)], *wR*(*F* ^2^), *S*	0.036, 0.088, 1.06	0.030, 0.072, 1.09	0.050, 0.108, 1.05
No. of reflections	4071	4457	6115
No. of parameters	263	263	388
Δρ_max_, Δρ_min_ (e Å^−3^)	0.34, −0.32	0.49, −0.71	0.32, −0.35

**Table:** 

	(V)	(VI*a*)	(VI*b*)
Crystal data			
Chemical formula	C_27_H_24_N_6_O	C_26_H_21_ClN_6_O	C_26_H_21_ClN_6_O
*M*_r_	448.52	468.94	468.94
*a*, *b*, *c* (Å)	10.2203 (15), 14.821 (2), 16.594 (2)	10.2298 (7), 14.8344 (9), 16.5321 (10)	9.6520 (8), 9.7408 (10), 14.1445 (12)
α, β, γ (°)	99.616 (5), 92.153 (6), 106.083 (5)	99.672 (2), 92.038 (2), 106.704 (2)	98.183 (4), 104.638 (3), 90.059 (4)
*V* (Å^3^)	2371.9 (6)	2359.4 (3)	1272.6 (2)
*Z*	4	4	2
μ (mm^−1^)	0.08	0.19	0.18
Crystal size (mm)	0.25 × 0.22 × 0.12	0.18 × 0.13 × 0.11	0.12 × 0.09 × 0.08

Data collection			
*T*_min_, *T*_max_	0.948, 0.990	0.926, 0.979	0.916, 0.986
No. of measured, independent and observed [*I* > 2σ(*I*)] reflections	116861, 10869, 8351	126128, 10821, 8240	60695, 5849, 4653
*R*_int_	0.074	0.079	0.074
(sin θ/λ)_max_ (Å^−1^)	0.650	0.650	0.651

Refinement			
*R*[*F* ^2^ > 2σ(*F* ^2^)], *wR*(*F* ^2^), *S*	0.050, 0.127, 1.06	0.058, 0.164, 1.04	0.050, 0.115, 1.03
No. of reflections	10869	10821	5849
No. of parameters	631	629	315
Δρ_max_, Δρ_min_ (e Å^−3^)	0.50, −0.23	0.74, −0.61	0.34, −0.53

Computer programs: *APEX3* (Bruker, 2018 ▸), *SAINT* (Bruker, 2017 ▸), *SHELXT2014* (Sheldrick, 2015*a* ▸), *SHELXL2014* (Sheldrick, 2015*b* ▸) and *PLATON* (Spek, 2020 ▸).

**Table 2:** Selected torsion angles (°) for inter­mediates (II) and (III)

Angle	(II)	(III)
N3—C4—N41—C42	−59.42 (19)	−59.7 (3)
N3—C4—N41—C47*A*	104.71 (15)	103.3 (2)
N41—C42—C71—C72	−34.0 (2)	−33.9 (3)
C5—C6—O61—C61	177.09 (17)	177.67 (17)

**Table 3:** Selected torsion angles (°) for products (IV)–(VI)

	(IV)	(V)	(V)	(VI*a*)	(VI*a*)	(VI*b*)
Angle	*x* = nil	*x* = 1	*x* = 2	*x* = 1	*x* = 2	*x* = nil
φ_1_	136.6 (2)	129.54 (18)	130.72 (18)	129.0 (2)	130.7 (2)	121.91 (18)
φ_2_	−58.1 (3)	−68.6 (2)	−70.2 (2)	−69.3 (3)	−72.1 (3)	−62.7 (2)
φ_3_	−15.6 (3)	−21.6 (3)	−25.7 (2)	−21.4 (3)	−26.0 (3)	−29.4 (3)
φ_4_	177.0 (2)	171.48 (17)	173.80 (18)	171.8 (2)	175.7 (2)	145.43 (18)
φ_5_	−4.3 (3)	−2.9 (3)	3.8 (3)	−4.9 (4)	0.4 (4)	−3.5 (3)
φ_6_	−176.38 (18)	−174.4 (2)	177.12 (17)	179.8 (3)	174.8 (2)	−177.71 (17)

Notes: φ_1_ represents the torsion angle N*x*3—C*x*4—N*x*41—C*x*42; φ_2_ represents the torsion angles N3—C4—N41—C47*A* in (IV) and (VI*b*), and C*x*4—N*x*41—N*x*41—C*x*7A in (V) and (VI*a*) (see Figs. 3 ▸–6 ▸ ▸ ▸); φ_3_ represents the torsion angle N*x*41—C*x*42—C*x*71—C*x*72; φ_4_ represents the torsion angle C*x*4—C*x*5—N*x*51—C*x*57; φ_5_ represents the torsion angle N*x*51—C*x*57—C*x*51—C*x*52; φ_6_ represents the torsion angle C*x*5—C*x*6—O*x*61—C*x*61.

**Table 4:** Hydrogen bonds and short intra­molecular contacts (Å, °) for com­pounds (II)–(VI) *Cg*1 and *Cg*2 represent the centroids of the C171–C176 and C271–C276 rings, respectively.

	*D*—H⋯*A*	*D*—H	H⋯*A*	*D*⋯*A*	*D*—H⋯*A*
(II)	N21—H21*A*⋯O81	0.90 (2)	2.10 (2)	2.989 (2)	171.1 (18)
	N21—H21*B*⋯O81^i^	0.86 (2)	2.094 (19)	2.8840 (18)	153.0 (18)
	N51—H51*A*⋯O61	0.85 (2)	2.41 (2)	2.7059 (17)	101.1 (17)
	N51—H51*B*⋯N41	0.90 (2)	2.571 (19)	2.888 (2)	101.5 (14)
	O81—H81*A*⋯N43^ii^	0.87 (2)	1.98 (2)	2.8506 (19)	178 (2)
	O81—H81*B*⋯N51^iii^	0.84 (2)	2.11 (2)	2.927 (2)	164 (2)
(III)	N21—H21*A*⋯O81	0.83 (3)	2.17 (3)	2.992 (3)	172 (3)
	N21—H21*B*⋯O81^i^	0.87 (3)	2.07 (3)	2.880 (3)	154 (2)
	N51—H51*A*⋯O61	0.91 (3)	2.41 (3)	2.706 (2)	98 (2)
	N51—H51*B*⋯N41	0.88 (3)	2.56 (3)	2.885 (3)	103 (2)
	O81—H81*A*⋯N43^ii^	0.83 (3)	2.03 (3)	2.862 (3)	178 (4)
	O81—H81*B*⋯N51^iii^	0.86 (3)	2.11 (3)	2.932 (3)	159 (3)
(IV)	N21—H21*A*⋯O81	0.89 (3)	1.94 (3)	2.826 (3)	173 (2)
	N21—H21*B*⋯N1^iv^	0.86 (3)	2.32 (3)	3.161 (3)	166 (3)
(V)	N121—H12*A*⋯N23	0.90 (2)	2.13 (2)	3.022 (2)	171.5 (19)
	N121—H12*B*⋯N243^v^	0.92 (2)	2.18 (2)	3.062 (2)	160 (2)
	N221—H22*A*⋯N13	0.84 (2)	2.19 (2)	3.023 (2)	173.4 (19)
	N221—H22*B*⋯N143^ii^	0.90 (2)	2.11 (2)	2.994 (2)	169 (2)
	C146—H146⋯N151^ii^	0.95	2.57	3.390 (3)	145
	C176—H176⋯N21^vi^	0.95	2.58	3.464 (2)	154
	C155—H155⋯*Cg*1^iv^	0.95	2.60	3.465 (2)	151
	C255—H255⋯*Cg*2^vii^	0.95	2.87	3.784 (2)	163
(VI*a*)	N121—H12*A*⋯N23	0.86 (3)	2.18 (3)	3.016 (3)	168 (3)
	N121—H12*B*⋯N243^v^	0.89 (3)	2.21 (3)	3.043 (3)	158 (3)
	N221—H22*A*⋯N13	0.78 (3)	2.23 (3)	3.012 (3)	173 (3)
	N221—H22*B*⋯N143^ii^	0.82 (3)	2.18 (3)	2.988 (3)	168 (3)
	C146—H146⋯N151^ii^	0.95	2.58	3.404 (4)	146
	C176—H176⋯N21^vi^	0.95	2.57	3.449 (3)	154
	C155—H155⋯*Cg*1^iv^	0.95	2.55	3.391 (3)	147
	C255—H255⋯*Cg*2^vii^	0.95	2.85	3.754 (3)	160
(VI*b*)	N21—H21*A*⋯N3^vi^	0.85 (2)	2.35 (2)	3.196 (2)	175.9 (19)
	N21—H21*B*⋯N43^viii^	0.90 (2)	2.08 (2)	2.946 (2)	163.6 (19)

Symmetry codes: (i) −*x* + 1, −*y* + 1, −*z*; (ii) −*x* + 1, −*y* + 1, −*z* + 1; (iii) −*x* + 1, −*y*, −*z*; (iv) −*x* + 1, −*y* + 2, −*z* + 1; (v) −*x* + 1, −*y* + 1, −*z* + 2; (vi) −*x*, −*y* + 1, −*z* + 1; (vii) −*x*, −*y*, −*z* + 2.
